# Rabies Outbreaks and Vaccination in Domestic Camels and Cattle in Northwest China

**DOI:** 10.1371/journal.pntd.0004890

**Published:** 2016-09-01

**Authors:** Ye Liu, He-Ping Zhang, Shou-Feng Zhang, Jin-Xiang Wang, Hai-Ning Zhou, Fei Zhang, Yu-Mei Wang, Long Ma, Nan Li, Rong-Liang Hu

**Affiliations:** 1 Laboratory of Epidemiology and Key Laboratory of Jilin Provincial Zoonosis Control and Prevention, Military Veterinary Research Institute, Academy of Military Medical Sciences, Changchun, China; 2 Ningxia Hui Autonomous Region Center for Animal Disease Control and Prevention, Yinchuan, China; Wistar Institute, UNITED STATES

## Abstract

In contrast to many countries where rabies has been well controlled in humans and livestock, even in wildlife, rabies is still endemic in almost regions of China. In Northwest China, rabies transmitted by stray dogs and wild foxes has caused heavy economic losses to local herdsmen, as well as causing numbers of human cases. In this study, as part of an investigation of ways to prevent rabies epidemics in livestock, we report an analysis of domestic cattle and camel rabies cases in Ningxia Hui (NHAR) and Inner Mongolia Autonomous Region (IMAR) and the immune efficacy of canine inactivated rabies vaccines in these animals. We found that rabies viruses from these animals are closely related to dog-hosted China I and fox-associated China III lineages, respectively, indicating that the infections originated from two different sources (dogs and wild foxes). As well as the previously reported Arctic and Arctic-related China IV lineage in IMAR, at least three separate phylogenetic groups of rabies virus consistently exist and spread throughout Northwest China. Since there is no licensed oral vaccine for wild foxes and no inactivated vaccine for large livestock, local canine inactivated vaccine products were used for emergency immunization of beef and milk cattle and bactrian (two-humped) camels in local farms. Compared with a single injection with one (low-efficacy) or three doses (high-cost), a single injection of a double dose of canine vaccine provided low-price and convenience for local veterinarians while inducing levels of virus neutralizing antibodies indicative of protection against rabies for at least 1 year in the cattle and camels. However, licensed vaccines for wildlife and large domestic animals are still needed in China.

## Author Summary

Rabies virus continues to cross carnivorous species and to infect humans and livestock in China. Rabies vaccination of the principal reservoir animals is even now being neglected in most regions of China, resulting in continuous expansion of rabies epidemics. Since there is no oral vaccine for stray dogs and wild animals and no inactivated vaccine for large domestic animals, rabies is not currently controlled in this country. We report rabies outbreaks caused by bites of dogs and wild foxes and the long-term effects on protection against rabies using canine inactivated vaccines in domestic camels and cattle. Our results indicate that at least three separate phylogenetic groups of rabies virus consistently exist and spread throughout Northwest China. Local canine vaccine products can be used to induce levels of virus neutralizing antibodies indicative of protection against rabies in cattle and camels; however, licensed oral and inactivated vaccines for reservoir carnivores and large domestic animals are urgently needed for elimination of rabies in China.

## Introduction

Rabies has been a continuous and serious threat to Chinese public health with three large epidemic waves since 1949 [1], reflecting the discontinuous effects of rabid animal control and prevention. During the latest epidemic wave (1996–present), the reported annual number of human rabies deaths has gradually decreased, to 744 in 2015 from a peak of 3,300 in 2007, mainly due to improvements in public awareness of rabies and the availability of human post-exposure prophylaxis (PEP) [2]. However, the rabies epidemic is still geographically expanding and new cases have been recorded in previously rabies-free and low incidence provinces such as Ningxia Hui Autonomous Region (NHAR), Qinghai, Gansu, and Tibet since 2011, because rabies control efforts in reservoir animals are even now being neglected in most regions of China [3].

In northwestern China, rabies transmitted by stray dogs and wild foxes has caused heavy economic losses to local herdsmen following infection of domestic animals such as cattle, camels, goats and horses [4,5], yet providing preventive vaccination to the herds and/ or reservoirs in these regions could prevent these losses. However, in China, as well as lacking an oral vaccine for the control of rabies in stray dogs and wild animals, no veterinary rabies vaccine has so far been developed or imported for domestic animals except owned dogs [6,7]. Although rabies prophylactic vaccination has been recommended for cattle by the World Organization for Animal Health (OIE), and successfully performed in rabies endemic countries [8], it is uncertain that emergency immunization using local canine rabies vaccine products has been able to block the spread of infection in ruminants. Here we report rabies outbreaks caused by bites of dogs and wild foxes and the long-term effects on protection against rabies using canine inactivated vaccines in domestic camels and cattle in NHAR and Inner Mongolia Autonomous Region (IMAR), China.

## Materials and Methods

### Ethics statement

All animal brain sampling was post-mortem. All animal experiments described in this paper have been conducted according to the Guidelines on the Humane Treatment of Laboratory Animals stipulated by the Ministry of Science and Technology of the People’s Republic of China and approved (license no. 2009–045) by the Animal Welfare Committee of the Military Veterinary Research Institute, Changchun, China. Rabies serological testing in our laboratory has been annually approved by ANSES-Nancy Laboratory (France) since 2013 (http://ec.europa.eu/food/animals/pet-movement/approved-labs/index_en.htm). No human patient-derived clinical materials and non-human primates were used in the completion of these studies.

### Epidemiological data

Epidemiological history of camel rabies cases caused by dog bites in NHAR was obtained from the local Center for Animal Disease Control and Prevention in April 2015. Background data on fox- and dog-associated cattle rabies in IMAR were collected by our laboratory in local farms from March 2013 to August 2015.

### Brain sample collection and assay

Brain tissues from clinically suspect and dead beef cattle (11 samples) and dairy cows (10 samples) were collected by our group from two farms in Urat Front Banner, Bayannur city, IMAR. Fifteen brain tissue samples of clinically suspect and dead camels were collected from a bactrian (two-humped) camel farm in Shahu Lake district, Yinchuan city, NHAR and sent to our laboratory for rabies diagnosis by the local Center for Animal Disease Control and Prevention. All brain samples were tested in our laboratory using the direct immunofluorescence assay (FDA), performed using LIGHT DIAGNOSTICS Rabies FITC-globulin conjugate (EMD Millipore Corporation, MA, USA) as described previously [9]. The complete rabies N gene in each sample was amplified through the reverse transcription polymerase chain reaction (RT-PCR) and sequenced as described previously [9]. Briefly, preparation of total RNA from brain tissue was conducted with TRIzol (Invitrogen Life Technologies, USA) according to the supplier’s instructions. One pair of primers covering the full length of the viral N cDNA was used as previously described [9]. PCR products were purified and nucleotide sequencing was performed on both forward and reverse strands of DNA fragments by Jilin Comate Bioscience Co., China. The sequences of the PCR products were submitted to GenBank, with accession numbers KM016901, KM016899, KU928249 and KU928250.

### Sequence alignment and phylogenetic analysis

A phylogenetic tree was constructed using the Maximum Likelihood method in MEGA 7.0.14 [10], in which the reliability of the phylogeny groupings was evaluated using bootstrapping with 1000 replicates. The general time-reversible model incorporating invariant sites and a GTR+I+c4 model was favored for all datasets. The percentage identities and similarity scores of the viral protein sequences were calculated using DNAstar Lasergene software. (DNASTAR, Inc., Madison, USA). Reference sequences (S1 Table) of the major lineages within China and worldwide were taken from GenBank except the WQ14 (accession no. KM016901), WQ14-RF (accession no. KM016899), WQ15 (accession no. KU928249) and NX15 (accession no. KU928250) strains reported in this study.

### Rabies vaccination in domestic camels and cattle

For emergency prophylaxis to prevent future outbreaks of rabies in the local farms, 300 adult cattle (270 beef cattle and 30 dairy cows) and 330 adult bactrian camels were randomly divided into 9 groups (Groups A–I, Table 1) and immunized intramuscularly with a single injection containing one, two, or three doses (1mL/dose, NIH potency 2.0IU) of canine inactivated vaccine produced by Jilin Heyuan Bioengineering Co. Ltd. The vaccine contains killed-rabies whole virus strain CVS-11 as antigen, 100 mg/ml aluminium hydroxide as adjuvant and 0.1 mg/ml thiomersal as a preservative.

**Table 1:** RVNA titres in cattle and two-humped camels after vaccination with rabies inactivated vaccine.

Animals	Groups	Doses of vaccine	RVNA after vaccination (mean ± SD, IU/mL)			
			3 months	6 months	9 months	12 months
Beef cattle	A	1	0.93±0.37	0.68±0.19	0	0
	B	2	6.07±1.65	4.61±1.25	2.87±0.47	0.84±0.32
	C	3	7.99±2.17	6.07±1.65	3.15±0.47	0.89±0.24
Dairy cattle	D	1	0.84±0.32	0.62±0.21	0	0
	E	2	5.44±0.82	3.78±0.62	2.37±0.39	0.77±0.33
	F	3	7.16±1.07	4.97±0.82	2.87±0.47	0.77±0.33
Camels	G	1	0.68±0.19	0.55±0.09	0	0
	H	2	4.61±1.25	2.93±0.83	1.32±0.57	0.67±0.19
	I	3	6.07±1.65	4.25±1.44	2.11±0.84	0.68±0.19

*p*^A,B^ < 0.05, *p*^D,E^ < 0.05, *p*^G,H^ < 0.05, *p*^B,C^ > 0.05, *p*^E,F^ > 0.05, *p*^H,I^ > 0.05.

Groups A–C contain 90 beef cattle (1 to 3-year old) each, groups D–F contain 10 dairy cows (1 to 3-year old) each, and groups G–I contain 110 camels (8 to 20-year old) each.

Blood samples were randomly collected from 45 cattle (30 beef cattle and 15 dairy cows) and 30 camels from the tail vein of cattle and the jugular vein of camels. Sera were separated by centrifugation following incubation at ambient temperature for 3h. Rabies virus neutralizing antibody (RVNA) was assayed using the standard FAVN method [11,12] before and once every 3 months after vaccination for 1 year. Variance analysis of RVNA titers was performed using SPSS 16.0 software for Windows (SPSS Inc., Chicago, IL, USA) to determine statistically significant differences in the quantitative data by one-way ANOVA. The results of the comparisons between groups were considered significantly different if *p* < 0.05. Data are expressed as mean ± SD.

### Accession numbers

The new N gene sequences in this study were submitted to GenBank, with accession numbers KM016901, KM016899, KU928249 and KU928250.

## Results

### Epidemiological background of rabies in domestic camels and cattle in Northwest China

According to the results of FDA and RT-PCR, all brain samples collected from 11 beef cattle, 15 camels and 10 dairy cows were diagnosed positive for rabies in our laboratory and the sequences of the viral N gene were absolutely identical to each other in individual farms.

Rabies in wild foxes was sporadically recorded in the early 1980s in the Xinjiang Uyghur Autonomous Region (XUAR) and IMAR, China [13]. In the spring of 2013, local farmers reported dozens of dead foxes and domestic animals in the pastures of XUAR and IMAR which were confirmed by laboratory test as dying of rabies strains highly similar to isolates from red foxes in Mongolia [4,5]. During February 2013–February 2014, a total of 26 sheep (Tacheng city), 2 camels and 1 beef cattle (Alxa Youqi County) died of rabies transmitted by wild foxes in XUAR and IMAR (S1 Fig) according to previous reports [4,5]. Another of the largest outbreaks of fox-associated rabies in domestic animals took place in March of 2014 in Urat Front Banner, Bayannur city, IMAR (S1 Fig). In one local farm, 11 beef cattle (1 to 3-year old) died of confirmed rabies transmitted by the bites of wild foxes. However, no rabies case was reported in other local farms at that time. Since 2015, following a steep decline in wild fox numbers as a result of rabies, no fox or fox-associated case has been identified in this region. Clearly, wild foxes and domestic animals should be considered for pre-exposure vaccination, not only to avoid financial losses or protection of wild animals, but because of their potential threat to human health.

In NHAR, no human case was reported before 2011, in which year there were 2 recorded rabies cases [3]. Following the growing epidemic of rabies, 8 human deaths caused by bites of rabid dogs were reported in 2013 in NHAR [3]. In April 2015, 15 bactrian camels (8 to 20-years old) in one farm in Shahu Lake district, Yinchuan city, NHAR (S1 Fig), died from confirmed rabies during a 20-day period. Early stage clinical symptoms included decreased appetite, cessation of rumination, excessive activity and agitation, later developing into paralysis, lip twitching, hypersalivation, tachypnea and howling, which lasted for 3–8 days. This semi-enclosed farm bred over 300 bactrian camels for tourist pleasure, in a compound in which other animals such as dogs, cats and wild carnivores could roam freely. Due to the absence of compulsory vaccination of animals in NHAR, rabies can be easily spread from rabid animals to camels; however, no rabid animals had been seen in recent years in this region. Similar animal cases were reported in a dairy cattle farm in Urat Front Banner, Bayannur city, IMAR, in which 10 dairy cows died from confirmed rabies in May 2015. The local people did not know which kind of animal transmitted the infection, or how they could prevent rabies introduction from elsewhere.

### Phylogenetic analysis of rabies isolates from Northwest China

**Fig 1:**
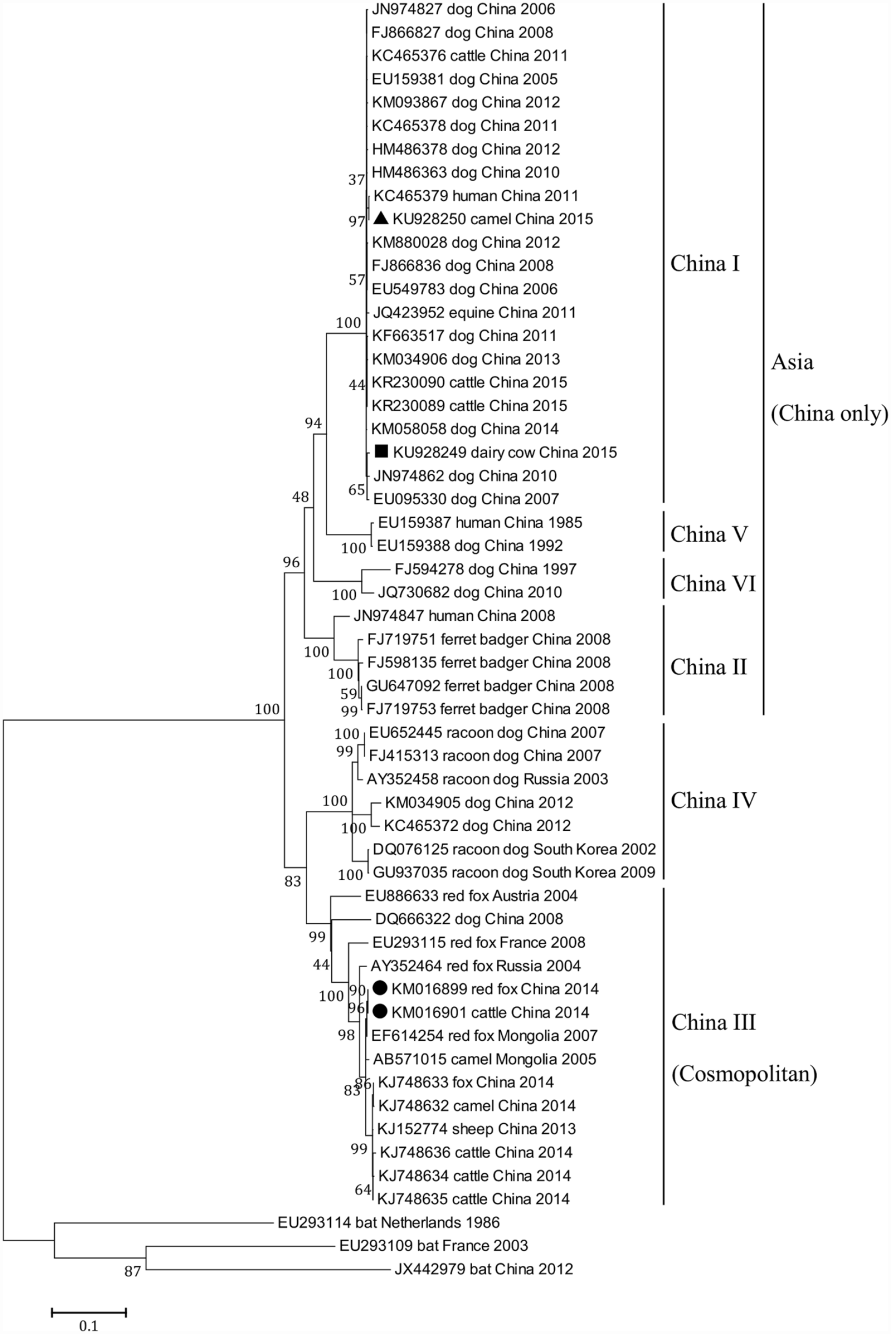
Maximum likelihood phylogenetic tree based on the complete rabies virus nucleoprotein gene. The tree is rooted with Irkut virus isolate JX442979, European bat lyssavirus type 1 and 2 isolates EU293109 and Eu293114. The RABV isolates in this study are marked using black triangles, black squares and black circles.

Phylogenetic analysis of the isolated viruses is an alternative way to track rabies transmission. Although all the collected samples were positive for rabies, the sequences of the rabies virus isolates from individual farms were absolutely identical to each other. Only one sequence from each farm was used in phylogenetic analysis. As shown in Fig 1, the camel (▲) and dairy cattle (■) cases were classified as China I, currently the main lineage originating from dogs nationwide and most similar to the dog-associated strains collected from IMAR and NHAR. This suggests that the observed cases resulted from the bites of rabid dogs. However, the strains in both animal species were distinct from isolates from wild fox-associated and Arctic related cases, indicating at least three separate phylogenetic groups in West China; i.e., China I, China III and IV (Fig 1). The rabies virus strains (●) isolated from wild fox and beef cattle by our laboratory clustered with China III (i.e. Cosmopolitan lineage) and were found to be highly similar to isolates from red foxes across countries in Eurasia. In China, most of wild fox-associated strains were collected during 2013–2014 in the northwestern regions of XUAR and IMAR [4,5] and distinct from the other five China lineages, suggesting that the introduction and spread of the wild fox rabies epidemic resulted in part from cross-border transmission between China and Mongolia and/ or other neighbouring countries [14,15].

### Rabies neutralizing antibody test after vaccination in domestic camels and cattle

**Fig 2:**
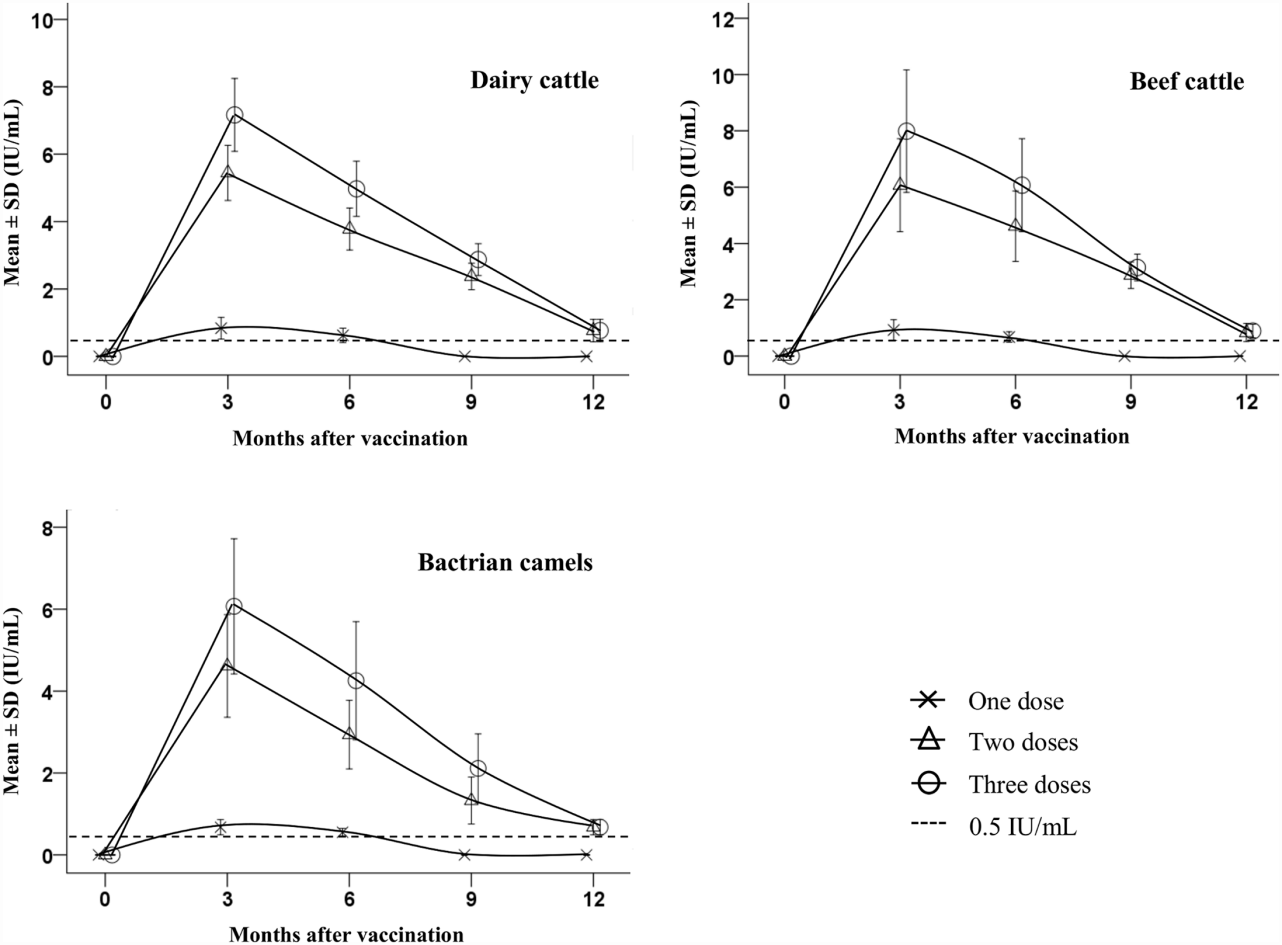
Rabies-neutralizing antibody response in cattle and bactrian camels after immunization with canine inactivated rabies vaccines. Error bars: mean ± SD.

The cut-off value of rabies RVNA titer, 0.50 IU/mL, is defined as the minimum antibody level affording complete protection [11]. No detectable RVNA was detected in any unvaccinated animal. In contrast, all animals in the random testing had RVNA titers after receiving a single vaccination. As shown in Table 1, there are significant differences (*p* < 0.05) in RVNA titers between the one-dose groups and multi-dose groups by one-way ANOVA, and no significant difference (*p* > 0.05) between two-dose groups and three-dose groups. Levels of RVNA indicative of protection in all tested animals vaccinated with two or three doses lasted more than one year (Fig 2); however, the RVNA levels in cattle were high relative to those in camels (*p* > 0.05), possibly due to genetic differences in the two animals. According to the local veterinary clinical records, no overt clinical reactions, such as allergic reactions, mortality, anorexia, pyrexia, rumination, changes in behaviour, weight gain, milk production, male and female fertility, occurred during the three-month period following immunization.

## Discussion

The ‘One health’ concept has been proposed as an effective way to successfully eliminate human rabies cases worldwide by 2030 [16]. In China, human rabies is a notifiable zoonosis through a comprehensive surveillance network; however, real-time surveillance of animal cases is still officially lacking. Up-to-date information about animal rabies, especially in wildlife, is generally difficult to obtain except in some cases involving human exposures. In light of the history of rabies epidemics, we should clearly recognise the serious situation of animal rabies control as requiring hard and long-term work in China and not mask the truth simply because of the current decrease in human rabies cases.

Molecular epidemiological studies on rabies viruses isolated in China indicate that the viruses can be divided into 6 lineages (China I–VI) [1], in which rabies cases in Northwest China in the last 10 years have all been caused by dog-associated China I and wildlife-associated China III and IV [3–5,17]. In the northwestern region of China, including NHAR, Gansu, XUAR and IMAR, there were hardly any reported human rabies cases before 2011 due to the sparse population, geographically remote regions and climatic extremes [3]. However, in recent years, following the implementation of the National Great Western Development and Strategy and projects relating to the conversion of degraded farmland into forests and grasslands, the recovery of the natural, social and economic environments have resulted in a significant increase in animal and human populations. It is likely, therefore, that rabies will rapidly spread among non-vaccinated animals and spill over into humans. There is also suggestion of a link between rabies cases in China and neighbouring countries: a sudden reappearance of fox-associated rabies at the Russian-Mongolian border in 2011–2012 was reported recently, in which the rabies virus isolates were almost identical to each other and very similar to the isolates from Mongolia, all belonging to the Cosmopolitan group (China III) [15].

Most livestock cases occurring in China as spillover from rabid carnivores have been reported as accidents in which vaccination of livestock was not performed to prevent re-emergence of rabies [5,17]. However, infected livestock, particularly cattle and camels, possibly with furious signs of rabies, provide a potential risk to veterinarians and handlers [8]. This underlines the urgency of implementation of rabies control measures for both humans and animals, especially stray dogs and wild foxes.

Currently, six local brands and four imported brands of canine inactivated vaccines have been approved by the Ministry of Agriculture of China, with sufficient production levels for rabies vaccination of domestic dogs. However, oral vaccines for stray dogs and wildlife remain at the laboratory-research level and for certain technical and official reasons, are not expected to be approved in China for production for another 5–10 years. In addition, as noted above, most large livestock cases occurring in China have been treated as occasional accidents in situations where inactivated vaccine for large animals is rarely used. There is no demand, with the result that inactivated rabies vaccine for large animals has not so far been developed and produced in China. Therefore, to control rabies in domestic animals, only one type of vaccine, i.e., canine inactivated vaccine, can be used to immunize via the intramuscular injection route. To be accepted by local veterinarians and farmers, the vaccine must be low-cost and easy to use. In this study, a single vaccination of two doses of canine vaccine has been shown to induce levels of virus neutralizing antibodies indicative of protection against rabies in cattle and camels; however, licensed vaccines for large domestic animals are still needed for use in pasture farms in China.

In endemic countries, the duration of immunity resulting from vaccination should be determined in the target animals and, according to the OIE recommendation [8], vaccines should confer protective immunity for at least 1 year. The cut-off value of rabies RVNA titer, 0.50 IU/mL, has been used to estimate the protective level after vaccination in this study; however, protection against RABV *in vivo* is complex, in which RVNA is only a partial contributor. It remains a challenge to determine how accurately the FAVN test, which utilizes *in vitro* neutralization for assaying RVNA, measures the protective level *in vivo* [18]. Therefore, there is a basic requirement to establish a new *in vitro* method for absolute proof of *in vivo* protection.

Camel rabies outbreaks have occurred mainly in the Middle East in the past few decades [19–20]; however, rabies vaccination in camels has hardly been reported. There are three subclasses of functional immunoglobulins (lgGs) present in the serum of Camelidae: lgG1 is a conventional heterotetrameric antibody, while lgG2 and lgG3 consist only of heavy-chain antibodies (HCAbs) [21]. Although the influence and effect of HCAbs on rabies vaccination *in vivo* and surveillance *in vitro* remain so far unknown, our data show that the current vaccine can confer protective immunity in camels for at least 1 year.

In conclusion, since vaccination and serological test programs for reservoir animals are the basic and effective approaches to prevent established and imported rabies in China, more research should be devoted to the development of oral vaccines for dogs and foxes. In addition, regular surveillance of cross-border transmission between China and neighbouring countries is required for wildlife rabies control.

## Supporting Information

S1 Fig Geographical distribution of rabies cases in the present study in Northwest China. Red dots indicate the location of rabies virus strains isolated from wild fox and cattle in the present study in IMAR. The green dot identifies camel rabies in the present study in NHAR. The black dots represent fox-associated rabies cases reported previously during the recent outbreaks in XUAR and IMAR.
(TIF)

S1 Table Rabies virus sequences used in the present study. (DOC)
