# Supplementary figures and images for: Rabies Outbreaks and Vaccination in Domestic Camels and Cattle in Northwest China
Source: PLoS Negl Trop Dis. 2016 Sep 1;10(9):e0004890. doi: 10.1371/journal.pntd.0004890 (PMC5008758; doi:10.1371/journal.pntd.0004890)

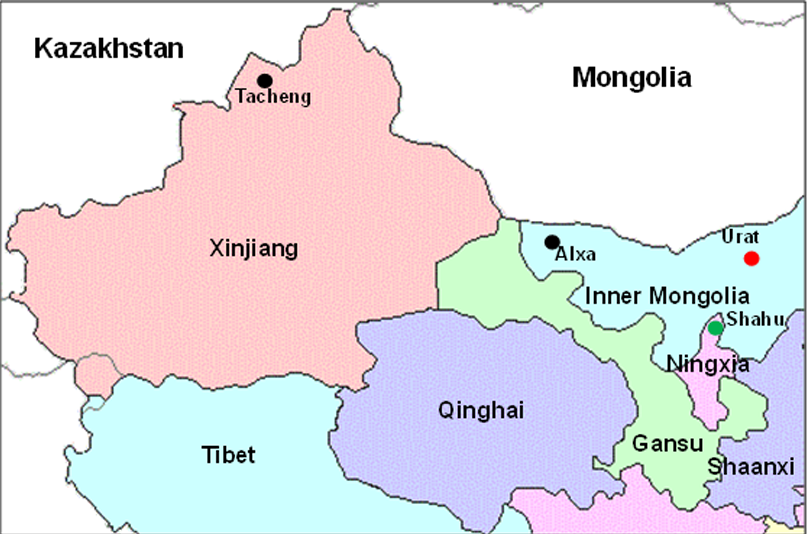

Supplement: S1 Fig — Red dots indicate the location of rabies virus strains isolated from wild fox and cattle in the present study in IMAR. The green dot identifies camel rabies in the present study in NHAR. The black dots represent fox-associated rabies cases reported previously during the recent outbreaks in XUAR and IMAR. (TIF) [file pntd.0004890.s001.tif]
