# Supplementary material for: Rabies Outbreaks and Vaccination in Domestic Camels and Cattle in Northwest China
Source: PLoS Negl Trop Dis. 2016 Sep 1;10(9):e0004890. doi: 10.1371/journal.pntd.0004890 (PMC5008758; doi:10.1371/journal.pntd.0004890)
Supplement: S1 Table — (DOC) [file pntd.0004890.s002.doc]

Table S1 Lyssavirus sequences used in the present study

| species | Strain | Isolation or origin | Year | GenBank no. |
| --- | --- | --- | --- | --- |
| RABV | CGX0606D | dog, China | 2006 | JN974827 |
|  | FJ010 | dog, China | 2008 | FJ866827 |
|  | CNM1101C | cattle, China | 2011 | KC465376 |
|  | JSL26 | dog, China | 2005 | EU159381 |
|  | BJ12-515 | dog, China | 2012 | KM093867 |
|  | CNM1104D | dog, China | 2011 | KC465378 |
|  | CSD0711D | dog, China | 2012 | HM486378 |
|  | CJS0634D | dog, China | 2010 | HM486363 |
|  | CNX1101H | human, China | 2011 | KC465379 |
|  | NX15 | camel, China | 2015 | KU928250 |
|  | DZ12 | dog, China | 2012 | KM880028 |
|  | FJ009 | dog, China | 2008 | FJ866836 |
|  | BD06 | dog, China | 2006 | EU549783 |
|  | BJ2011E | equine, China | 2011 | JQ423952 |
|  | ShaanxiRab007 | dog, China | 2011 | KF663517 |
|  | CGS1301D | dog, China | 2013 | KM034906 |
|  | SXYL15 | cattle, China | 2015 | KR230090 |
|  | SXBJ15 | cattle, China | 2015 | KR230089 |
|  | NB14 | dog, China | 2014 | KM058058 |
|  | WQ15 | dairy cow, China | 2015 | KU928249 |
|  | CSC1014D | dog, China | 2010 | JN974862 |
|  | Yunnan_Md06 | dog, China | 2007 | EU095330 |
|  | J | human, China | 1985 | EU159387 |
|  | CQ92 | dog, China | 1992 | EU159388 |
|  | N11 | dog, China | 1997 | FJ594278 |
|  | CYN1009D | dog, China | 2010 | JQ730682 |
|  | CSH0501D | dog, China | 2008 | JN974874 |
|  | JX08-47 | ferret badger, China | 2008 | FJ719751 |
|  | ZJ-LA | ferret badger, China | 2008 | FJ598135 |
|  | JX08-45 | ferret badger, China | 2008 | GU647092 |
|  | JX08-48 | ferret badger, China | 2008 | FJ719753 |
|  | NeiMeng1025B | raccoon dog, China | 2007 | EU652445 |
|  | NeiMeng925 | raccoon dog, China | 2007 | FJ415313 |
|  | 857r | raccoon dog, China | 2003 | AY352458 |
|  | CQH1202D | dog, China | 2012 | KM034905 |
|  | CXZ1201D | dog, China | 2012 | KC465372 |
|  | SKRDG0204CW | raccoon dog, South Korea | 2002 | DQ076125 |
|  | KRVR0906 | raccoon dog, South Korea | 2009 | GU937035 |
|  | Collection-date 2004 | red fox, Austria | 2004 | EU886633 |
|  | Jiangsu_Yc63 | dog, China | 2008 | DQ666322 |
|  | 9147FRA | red fox, France | 2008 | EU293115 |
|  | RV257 | red fox, Russia | 2004 | AY352464 |
|  | WQ14-RF | red fox, China | 2014 | KM016899 |
|  | WQ14 | cattle, China | 2014 | KM016901 |
|  | Mongolia 6 | red fox, Mongolia | 2007 | EF614254 |
|  | MGL-33 | camel, Mongolia | 2005 | AB571015 |
|  | NMFOX01 | fox, China | 2014 | KJ748633 |
|  | NMCAM02 | camel, China | 2014 | KJ748632 |
|  | NMSH01 | sheep, China | 2013 | KJ152774 |
|  | NMC04 | cattle, China | 2014 | KJ748636 |
|  | NMC02 | cattle, China | 2014 | KJ748634 |
|  | NMC03 | cattle, China | 2014 | KJ748635 |
| EBLV-1 | 03002FRA | bat, France | 2003 | EU293109 |
| EBLV-2 | 9018HOL | bat, Netherland | 1986 | EU293114 |
| IRKV | IRKV-THChina12 | bat, China | 2012 | JX442979 |
